# Evaluation of E-Health Applications for Paediatric Patients with Refractory Epilepsy and Maintained on Ketogenic Diet

**DOI:** 10.3390/nu13041240

**Published:** 2021-04-09

**Authors:** Anna-Maria Costa, Maddalena Marchiò, Giulia Bruni, Silvia Maria Bernabei, Silvia Cavalieri, Marina Bondi, Giuseppe Biagini

**Affiliations:** 1 Laboratory of Experimental Epileptology, Department of Biomedical, Metabolic and Neural Sciences, University of Modena and Reggio Emilia, 41125 Modena, Italy; annamaria.costa@unimore.it (A.-M.C.); gbiagini@unimore.it (G.B.); 2 AOU Meyer Hospital Florence, 50139 Florence, Italy; giulia.bruni@meyer.it; 3 Department of Paediatric Specialties and Liver, Kidney Transplant, UO Nutritional Rehabilitation, “Bambino Gesù” Children’s Hospital, 00165 Rome, Italy; silviamaria.bernabei@opbg.net; 4 Department of Foreign Languages and Literatures, University of Verona, 37129 Verona, Italy; silvia.cavalieri@univr.it; 5 Department of Studies on Language and Culture, University of Modena and Reggio Emilia, 41121 Modena, Italy; marina.bondi@unimore.it; 6 Centre for Neuroscience and Neurotechnology, University of Modena and Reggio Emilia, 41125 Modena, Italy

**Keywords:** COVID-19 pandemic, dietary management, drug-resistant epilepsy, E-health applications, Italy, ketogenic diet, smart technology, smartphone app

## Abstract

E-health technologies improve healthcare quality and disease management. The aim of this study was to develop a ketogenic diet management app as well as a website about this dietary treatment and to evaluate the benefits of giving caregivers access to various web materials designed for paediatric patients with refractory epilepsy. Forty families participated in the questionnaire survey, from January 2016 to March 2016. All caregivers were exposed to paper-based materials about the ketogenic diet, whereas only 22 received the app, called KetApp, and videos produced by dieticians. Caregivers with free access to web materials were more satisfied than the others with the informative material provided by the centre (*p ≤* 0.001, Mann–Whitney test). Indeed, they showed a better attitude towards treatment, and they became more aware of dietary management in comparison to the control group (*p ≤* 0.001). Moreover, caregivers provided with web materials were stimulated to pursue the treatment (*p =* 0.002) and to introduce it to their children and other people (*p =* 0.001). Additionally, caregivers supplied with web materials were more willing to help other families in choosing the ketogenic diet (*p =* 0.004). Overall, these findings indicate that web materials are beneficial for caregivers of paediatric patients with refractory epilepsy in our centres. Thus, the use of e-health applications could be a promising tool in the daily aspects of ketogenic diet management, and it is especially of value in the attempt to start or maintain the diet during the ongoing COVID-19 pandemic crisis.

## 1. Introduction

E-health applications are based on the use of information and communications technologies in support of health-related fields, such as healthcare services, health surveillance, health literature, knowledge, and research [1]. These tools have the potential to remarkably enhance health service efficiency, accessibility and delivery to thousands of patients, as well as to improve patient outcomes [1,2]. For this reason, the use of e-health technologies to provide innovative solutions for disease management has raised patients’, caregivers’, and health professionals’ hopes [2].

In recent years, the use of instant messaging services [3,4], the development of a large variety of smartphone applications (apps) [2,5,6] and online platforms (e.g., Zoom, Polycom, or Doximity) [7] have led to improved communication within medical teams and family caregivers by simplifying information sharing, early treatment initiation, and therapy management. During the ongoing COVID-19 pandemic, e-health applications have been intensively used to reduce the risk of cross-contamination caused by close contact. Indeed, the adoption of e-health solutions has been remarkably important to continue providing knowledge and information to patients and caregivers while attempting to “flatten the curve” of the increasing number of COVID-19 cases [8]. Interestingly, smartphone apps are appreciated because they are easily accessible, acceptable, and able to support social distancing efforts [8]. Concerning the development of apps, some of them seem to be designed for healthcare professionals, whereas others are most suitable as patient-centred apps. Furthermore, some apps can cover a wide range of clinical knowledge, and others can be tailored to specific diseases/disorders [2,9]. For instance, apps for epileptic seizure self-management have been employed in Australia and the United States [10,11]. Also, smartphone apps for seizure self-management would be well-accepted by patients with epilepsy in China [5].

In general, particular attention must be paid to the management of treatments for drug-resistant epilepsy. For instance, the ketogenic diet is a high-fat, low-carbohydrate and normal protein diet and is used to control antiseizure drug-refractory epilepsy [12,13]. Indeed, the classical 4:1 ketogenic diet seems to be effective in up to 55% of children with refractory seizures after three months [14]. During the coronavirus outbreak, the management of children with drug-resistant epilepsy on ketogenic diet therapies through telemedicine has been demonstrated to be feasible and well-received by the families and patients in Argentina [3] and the United States [7]. In Italy, specific guidelines and therapeutic approaches for the care of patients undergoing a ketogenic diet, especially in an acute medical setting, should be improved [15], as the introduction of this specific treatment requires an abrupt change of eating habits and a constant monitoring of possible side effects [16,17,18]. Indeed, the ketogenic diet aims at switching the brain metabolism from glucose dependence to the utilisation of ketone bodies. For this reason, it requires a limited consumption of cereal-based foods and a reduction in fruit and vegetable intake. In this dietary treatment, the low variability, palatability and tolerability can be compensated by food products commercialised by specific food companies. Similarly, side effects, such as gastrointestinal disorders, nephrolithiasis, growth retardation, hyperlipidaemia and mineral and vitamin deficiency should be avoided with the help of health professionals [19]. Notably, even if e-health technologies and the use of remote monitoring by telecommunications have recently increased in Italy [16,20], adequate information for long-term management of the ketogenic diet cannot be easily found by patients and caregivers [21]. For this reason, patients and caregivers’ free access to different e-health resources should play a key role in the dietary management of drug-resistant epilepsy.

Here, we evaluate family caregivers’ satisfaction to assess the feasibility and acceptability in our centres of using different web materials, including smartphone apps, websites and videos, for the management of paediatric patients with refractory epilepsy and maintained on a ketogenic diet. Specifically, to assess the beneficial effects of web materials specifically designed for caregivers and paediatric patients with a diagnosis of refractory epilepsy and treated with the ketogenic diet, we carried out an analysis aimed to compare the value of paper-based informative materials and web-based ones.

## 2. Materials and Methods

### 2.1. Patients

We considered a cohort of paediatric patients with a diagnosis of refractory epilepsy still treated at the time of the survey or, alternatively, treated in the past with the ketogenic diet. Particularly, children and their families were recruited in the Policlinico Hospital (Modena), in the Meyer Hospital (Florence), and in the “Bambino Gesù” Children’s Hospital (Rome). The inclusion criteria were a confirmed diagnosis of epilepsy, refractoriness to antiepileptic drugs, age ranging from 0 to 17 years, and informed consent signed by parents. At variance, exclusion criteria were acute or chronic metabolic diseases unrelated to epilepsy and the lack of adherence to the nutritional protocol. Particularly, the lack of adherence to the nutritional protocol was assessed by interviews during the routine checkup and phone calls. Moreover, the lack of adherence was also assessed by monitoring the blood glucose value (mg/dL), as well as the ketosis (mmol/L) and seizure occurrence during the routine checkup and/or by means of the smartphone app named KetApp. Specifically, the final ratio of lipids and proteins + carbohydrates that patients should reach was 2/4:1, and the dietary treatment also needed to be monitored and supplemented as previously detailed [17]. The Ethics Committee of Modena (4206/C.E.) approved the research protocol according to local regulations, and informed written consent was obtained from relatives of patients.

### 2.2. Caregivers

Caregivers were defined as the family members (e.g., parents and grandparents) or other family delegates (e.g., nannies), who were primarily responsible for providing everyday care for the child [22]. Indeed, caregivers were directly involved in the management of the ketogenic diet, such as cooking meals, and they oversaw the strict dietary rules of the treatment.

### 2.3. Questionnaire

The questionnaire followed the structure of the Information Satisfaction Questionnaire (ISQ) [23] and aimed at evaluating the quality of the information under examination considering comprehensiveness, accuracy, credibility, relevance, and suitability. The questionnaire was written using simple language, as was suggested in the guidelines provided by the working group for health literacy of the health section of Emilia-Romagna Region. The questionnaire was completed by volunteer caregivers from January 2016 to March 2016. As reported in our previous study [21], the aim was to evaluate their perception of and satisfaction with the informative materials regarding the ketogenic diet. The questionnaire was written using a simple language and it was anonymous [21]. The questionnaire was detailed in our previous study [21], and it was divided into four sections with the following titles: (1) General information; (2) Where did you find preliminary information about the ketogenic diet? (3) Can you evaluate the informative materials on the ketogenic diet given by the centre that follows your child? (4) Suggestions to improve the information for families (see supplementary material). Except for the last section, the questionnaire comprised multiple-choice questions with a 5-point Likert that ranged from 1 (strongly disagree) to 5 (strongly agree). To compare the efficacy of paper-based materials with web-based ones, we decided to improve the analysis of the third section that was divided into four sub-sections: (i) booklets/leaflets, (ii) website, (iii) app, and (iv) the overall material [21]. Parents were asked only to complete the sub-sections dealing with the informative materials they were provided.

### 2.4. Booklets

The booklets represented paper-based information, consisting of an average of 30 pages with a combination of text and images. One booklet was produced by the medical nutrition company “Nutricia”, whereas the other two were produced by the participating hospital centres [21]. The booklets were distributed during doctor–caregiver encounters by health operators.

### 2.5. Website

The website was created specifically for this research in 2016. Particularly, the first version of the website, available in 2016, was accessible only using a username and password provided by the dietician. In 2020, we implemented our Italian website created by health professionals, and then the second version of the website (www.dietachetogenica.unimore.it) became fully accessible.

### 2.6. App

KetApp is a tool, created by the University of Modena and Reggio Emilia, designed to assist nutritional compliance for all patients on a ketogenic diet. Twenty-two caregivers were given access to the pre-release version of the app, through usernames and passwords provided by the dietician. Data collected by the app were output in Word and Excel, and then sent by email.

### 2.7. Videos

The videos of the Keto recipes were directly created by the dietician of the Policlinico Hospital (Modena). The videos were reviewed during doctor–caregiver encounters, to explain how to follow a ketogenic diet in an interactive way.

### 2.8. Statistical Analysis

Data were analysed using Sigma Plot 11 (Systat Software, San Jose, CA, USA). The group that was given access only to the paper-based materials and the group that also had access to web-based materials were compared by the Mann–Whitney test. Results are shown as medians and interquartile ranges and considered significant at *p* < 0.05.

## 3. Results

### 3.1. Characterisation of Paediatric Patients and Their Families

A total of 40 paediatric patients and their families participated to our study. Particularly, 33 children and their families were recruited in the Policlinico Hospital, five in the Meyer Hospital, and two in the “Bambino Gesù” Children’s Hospital. According to our previous study, in which we presented a detailed characterisation of the patients and their caregivers [21], paediatric patients were mainly represented by girls (65.0%) with an age ranging from 4 to 6 years (42.5%). At variance, most of the caregivers were mothers (60.0%) of Italian nationality (77.5%). Generally, caregivers ranged in age from 30 to 40 (60.0%) and held a junior high school degree (40.0%). Moreover, 90.0% of caregivers could access the Internet. However, 52.1% found the preliminary information about the dietary therapy only through health institutions.

At the time of the questionnaire (2016), the majority of children were still treated with the ketogenic diet (62.5%) and had been following the treatment plan for two years on average. In the current year (2021), only five families and their children are still using the web materials since they completed the questionnaire in 2016.

All caregivers had free access to paper-based informative materials, and they considered booklets readable (65.0% of 4 = I agree) and understandable (60.0% of 4 = I agree). However, they also observed that booklets were neither satisfactory nor adequate to make an informed choice (62.5% of 2 = I disagree) and to explain the ketogenic diet to other people who might be interested in it (57.5% of 2 = I disagree, and 25.0% of 1 = I strongly disagree) [21].

Notably, foreign caregivers with difficulty in understanding Italian asked for translated versions of informative materials, or at least with easier language, and to provide ethnic recipes respecting cultural differences in the use of food [21].

### 3.2. The Design of an Italian Website about the Ketogenic Diet

The website called “www.dietachetogenica.unimore.it” provided information about the ketogenic diet, and the menu of the website was divided into six different sections. The first section concerns the diet: (i) history; (ii) mechanism of action; (iii) therapeutic implications in several diseases (e.g., glucose transporter type 1 deficiency syndrome, pyruvate dehydrogenase deficiency, obesity, Dravet syndrome, West syndrome, Lennox–Gastaut syndrome, migraine, diabetes, Parkinson’s disease, Alzheimer’s disease, amyotrophic lateral sclerosis, ageing, and brain tumours); (iv) contraindications; (v) clinical monitoring; (vi) variations to the classic protocol (e.g., the modified Atkins diet, the medium-chain triglyceride diet, and the low glycaemic diet); (vii) ketogenic artificial nutrition; (viii) monitoring of side effects; (ix) allowed food; (x) hidden sources of carbohydrates; (xi) first ketogenic grocery shopping; (xii) food labels; (xiii) ketogenic starter kit; (xiv) ketogenic diet at home; (xv) ketogenic diet at school; (xvi) flavours and frequencies of consumption. The second section regards epilepsy, epileptic seizures, antiepileptic treatment, and pharmacological tolerability. Finally, the third section focuses on the autosomal dominant polycystic kidney disease, giving an overview on the tolvaptan treatment and new treatment prospects. The last sections concern information about the article and publications, app, and research group.

### 3.3. The Design of an Italian App for the Management of the Ketogenic Diet

KetApp was primarily developed as an Italian pre-release version, which was strongly supported by health professionals as a way to help caregivers after the release of children who were affected by drug-resistant epilepsy from the Policlinico Hospital (Modena).

The architecture was designed to be easy to understand. The app allows to: (i) record diet parameters (e.g., daily intake of macro- and micro-nutrients, daily kilocalories and ratios agreed upon with the dietician); (ii) generate daily and weekly receipts and menus; (iii) record blood glucose and ketosis values; (iii) record the anthropometric data (e.g., weight and height) and monitor them over time; (iv) record epileptic seizure data (e.g., day, hour, duration, description of the seizures, and all actions taken).

**Figure 1:**
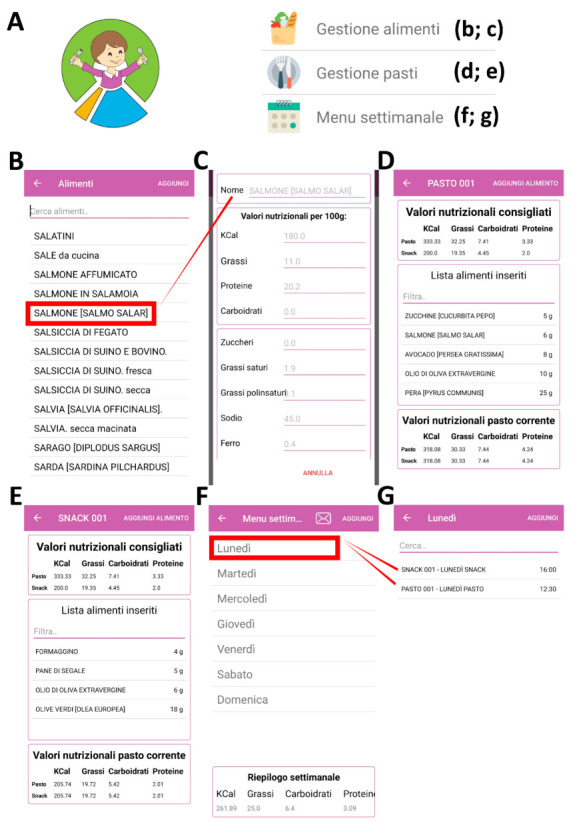
Using the app to learn about the nutritional facts of each food and create new daily or weekly menus. The logo and the first part of the drop-down menu are reported in (**A**). An example of food management (“gestione alimenti”) is reported in (**B**,**C**). An example of meal management (“gestione pasti”) is reported in (**D**,**E**). Particularly, in the section dedicated to meal management, the suggested nutritional values (“valori nutrizionali consigliati”) are above the list of inserted food, whereas the selected nutritional values (“valori nutrizionali pasto corrente”) are placed under the list. An example of a weekly menu (“menu settimanale”) with a weekly summary (“riepilogo settimanale”) is reported in (**F**,**G**).

In the following figures, the values refer to a child undergoing, for example, a dietary therapy (1000 Kcal/day) with a final ratio of lipids and proteins + carbohydrates equal to 3:1. In Figure 1, we report an example of how to learn about the nutritional facts of each food and create new daily or weekly menus. Particularly, the first option of the drop-down menu refers to food management (“gestione alimenti”). By clicking on this option (Figure 1A), it is possible to search for different types of food (e.g., salmon), and their nutritional values, per 100 g (Figure 1B,C). Then, it is possible to manage the meals (“gestione pasti”) and create new recipes by choosing different foods from the list. Both main meals and snacks should agree with the daily kilocalories and ratios approved by the dietician (Figure 1D,E). Once a new main meal (e.g., code: PASTO 001) or snack (e.g., code: SNACK 001) has been created, it is possible to use it in the weekly menu (“menu settimanale”). For instance, “PASTO 001” and “SNACK 001” were used in the menu for Monday (Figure 1F,G).

**Figure 2:**
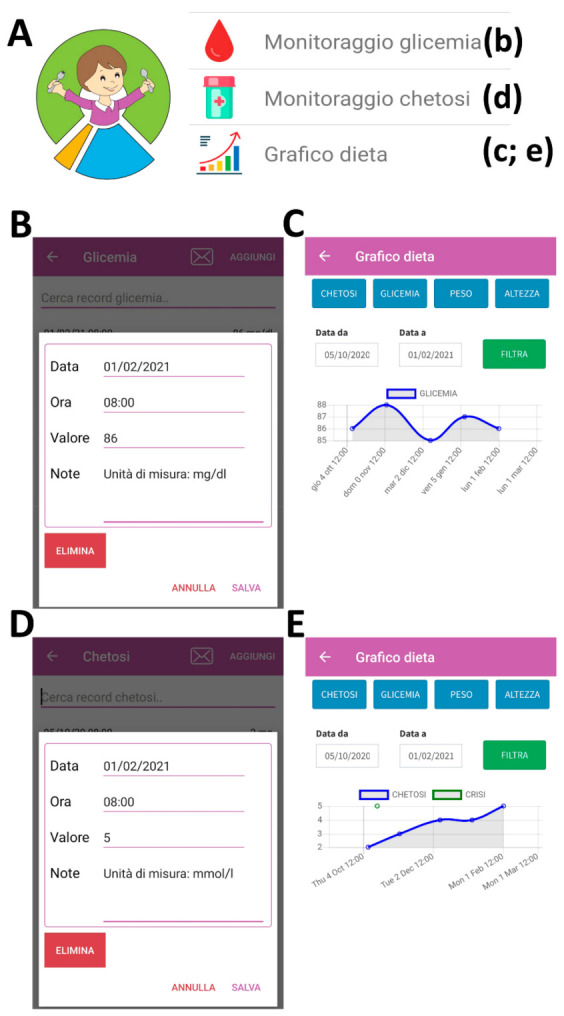
Using the app to record blood glucose and ketosis values. The logo and the second part of the drop-down menu are reported in (**A**). An example of recording of the blood glucose value (mg/dl) is reported in (**B**,**C**). An example of recording of the ketosis value (mmol/L) is reported in (**D**,**E**).

In Figure 2, we report a usage example to record blood glucose (“monitoraggio glicemia”) and ketosis values (“monitoraggio chetosi”). Indeed, for both parameter options (Figure 2A), it is possible to record the day and hour of measurement (Figure 2B,D) and monitor them over time with a graph (“grafico dieta”; Figure 2C,E).

**Figure 3:**
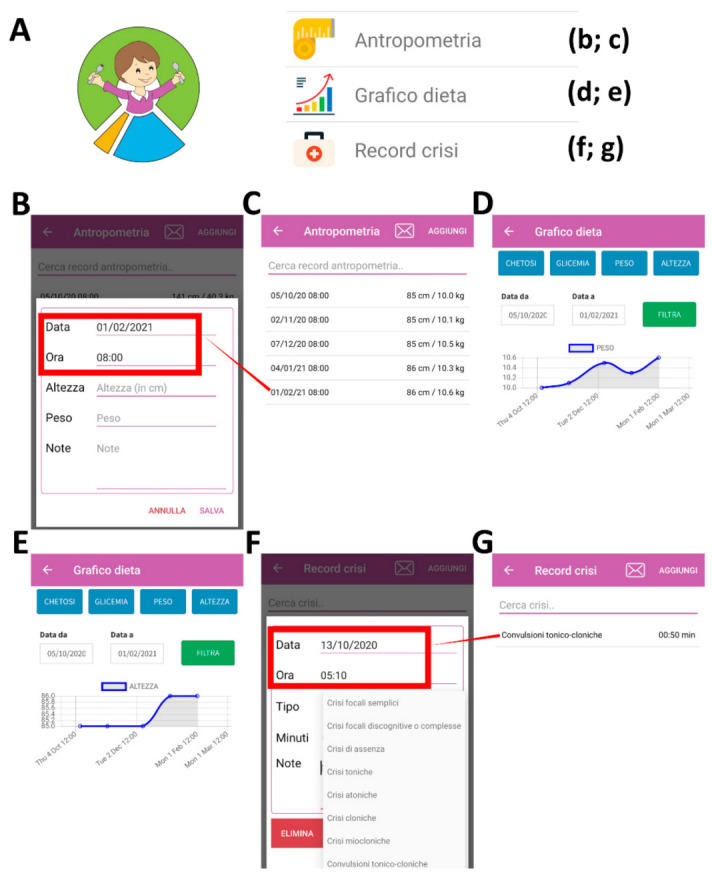
Using the app to record the anthropometric data and seizure values. The logo and the third part of the drop-down menu are reported in (**A**). An example of recording of the anthropometric data is reported in (**B**–**E**). An example of recording of the seizure values is reported in (**F**,**G**).

In Figure 3, we show how to record the anthropometric data (“antropometria”) and the number and type of seizures (“record crisi”) over time (Figure 3A). In this regard, both weight (“peso”) and height (“altezza”) parameters can be recorded in the app (Figure 3B–E). The different types of seizures (e.g., focal with or without impaired awareness, absence, tonic, atonic, clonic, myoclonic, or tonic–clonic) can also be reported (Figure 3F,G) and monitor over time with a graph (“grafico dieta”; Figure 2E).

### 3.4. Different Channels of Communication through Which Caregivers Had Received Information about the Ketogenic Diet

**Figure 4:**
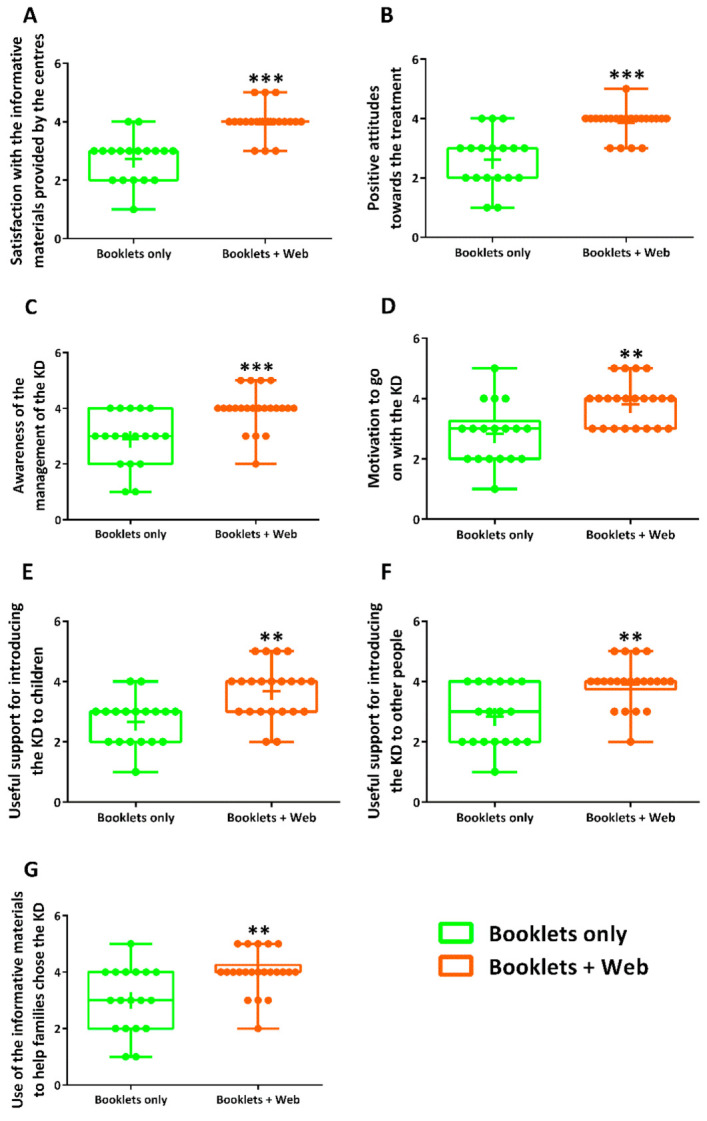
Comparison between channels of communication through which caregivers received information about the ketogenic diet. The group called “Booklets only” included caregivers with access only to booklets/leaflets, whereas the group known as “Booklets + Web” consisted of caregivers with access also to the KetApp and videos. Specifically, the two groups were compared in relation to (**A**) the satisfaction about the level of information of the materials provided by the centres; (**B**) the change in the attitude on the treatment after reading the informative materials; (**C**) the awareness of the management of the ketogenic diet; (**D**) the motivation to go on with the ketogenic diet; (**E**,**F**) the usefulness of the informative materials to explain the diet to children and other people involved in the dietary management; (**G**) the usefulness of the informative materials in motivating other families to choose the ketogenic diet (** *p* < 0.01, *** *p* < 0.001; Mann–Whitney test). The whiskers of the boxes represent the minimum and maximum values. In addition to the values of median, the mean is also represented by a +. KD, Ketogenic diet.

The results of the questionnaires, with the characterisations of the caregivers who were involved in the survey and a detailed analysis of the informative booklets, were presented in our previous study [21]. Here, we focus on a comparison between the group that was given access only to the booklets (*n* = 18) and the group that also had access to different web materials about the ketogenic diet (*n* = 22). All the caregivers were asked about their overall satisfaction with the informative material that they received from the medical centre following their child. Notably, the website was not fully available in 2016, so no caregivers were asked to complete the sub-sections of the questionnaire regarding the website. Interestingly, the caregivers with access to the web-based materials were significantly more satisfied than those without any access (Booklets only vs. Booklets + Web; 3.0, 2.0–3.0 vs. 4.0, 4.0–4.0; *p ≤* 0.001; Figure 4A). Access to the information on the app and the videos significantly increased caregivers’ positive attitudes towards the treatment, in comparison to the attitude of caregivers with access only to the booklets (3.0, 2.0–3.0 vs. 4.0, 4.0–4.0; *p ≤* 0.001; Figure 4B). The group with access to the web-based materials also became more aware of the management of the diet, in comparison to those without access to that kind of material (3.0, 2.0–4.0 vs. 4.0, 4.0–4.0; *p ≤* 0.001; Figure 4C). Moreover, caregivers with access to the app and the videos were significantly more motivated by the informative materials to go on with the diet, in comparison to the other participants in the survey (3.0, 2.0–3.3 vs. 4.0, 3.0–4.0; *p =* 0.002; Figure 4D). In comparison to the control group, caregivers with access to web materials about the ketogenic diet significantly thought that the information they received had helped them to explain the ketogenic diet to their children (3.0, 2.0–3.0 vs. 4.0, 3.0–4.0; *p =* 0.001; Figure 4E) and other people involved in the management of the diet (3.0, 2.0–4.0 vs. 4.0, 3.8–4.0; *p =* 0.001; Figure 4F). Finally, caregivers with access to the app were significantly more inclined to use the informative materials to help another family choose the ketogenic diet than those without any access (3.0, 2.0–4.0 vs. 4.0, 4.0–4.3; *p =* 0.004; Figure 4G).

## 4. Discussion

This study aimed to assess the efficacy of giving caregivers access to e-health technologies for the management of epileptic children receiving a ketogenic diet in our Italian centres. Specifically, the analysis was based on a questionnaire survey [21], which had at least the following advantages: (i) questionnaires were anonymous and caregivers felt free to express their opinions; and (ii) questionnaires were more economic, faster, and were considered more voluntary than interviews [3,24]. Our study demonstrated that the use of a website about the ketogenic diet specially designed by health professionals, the Italian app “KetApp”, and some videos of Keto recipes created by dieticians increased the satisfaction of caregivers and their positive attitude towards the dietary treatment of drug-resistant epilepsy. None of the caregivers stated that they strongly disagreed with the positive contribution made by e-health technologies in promoting the continuation of the ketogenic diet. Most caregivers would support the use of web-based materials, in addition to booklets and leaflets, to introduce the treatment to children and other people involved in the management of the diet. Eighty-two percent of the caregivers would recommend the therapeutic treatment of drug-resistant epilepsy with the ketogenic diet after using e-health technologies.

The application of a ketogenic diet is suitable for children and young infants in paediatric intensive care units for neurological disorders. Indeed, it is mainly used in cases of chronic refractoriness to antiepileptic drugs, but it is also a therapeutic option for the acute stage of refractory/super-refractory status epilepticus [25]. The use of this dietary treatment is challenging because of the critical status of the patients [25] and the low variability, palatability, and tolerability of the diet itself [19]. Furthermore, a ketogenic diet should be started as soon as possible, and an optimal management of the paediatric patients receiving this dietary therapy is required from the beginning [26]. When conducted properly, a ketogenic diet is tolerated [27] and it can induce an increased number of patients achieving seizure freedom over time [14,28]. Then, a correct management of the ketogenic diet is also important to avoid side effects [16,17,19], controversies [29], and, in some cases, a phenomenon known as the rebound effect [30], which is characterised by a paradoxical seizure worsening [31]. Thus, the initiation and maintenance of the dietary treatment are the result of concomitant efforts of paediatric neurologists/epileptologists, dieticians, families and other patients’ caregivers [32].

Nowadays, apps are innovative tools to manage and store patients’ records [16]. Through a smart device, it is possible to collect a huge amount of information quickly, and to represent the data of interest using tables, graphs, or charts to promote easy interpretation [2]. Recently, it was demonstrated that an automatic ketogenic diet planning application could decrease the burden of hand computation, offer an extensive variety of food choices, and help the users follow the therapeutic treatment recommended by the dietician [33]. Then, the importance of e-health technologies was also clear at the beginning of the COVID-19 pandemic, when a large variety of digital applications, processes, and platforms were adopted to continue providing knowledge to patients and caregivers, without affecting the social distancing efforts [8]. In Argentina, an interesting patient satisfaction survey demonstrated for the first time that caregivers were pleased with the management of the dietary treatment through WhatsApp, which could represent a simple but comprehensive tool to manage the ketogenic diet in patients living far from the treating centre or during difficult conditions, such as the ongoing crisis [3]. According to our survey, we also demonstrated the importance of the mobile applications in the management of the dietary treatment in our centres and its usefulness in maximising the success of the ketogenic diet.

Moreover, specific videos and websites designed by health professionals could become additional instruments for providing informative material about dietary management strategies. Indeed, it was reported that most caregivers and patients preferred to gather information about their diseases and treatment alternatives from different sources, such as from their healthcare providers, other patients or families, and on the Internet [34]. However, the web presents a wide range of information, some of which is erroneous. Misleading information could increase, in particular, the risks of improper self-diagnosis, the use of damaging therapeutic attempts, and the delay of visits to health professionals. Given the extensive use of the Internet and its remarkable impact, it was suggested that health websites might be designed to provide accurate information, helping patients and their families make better healthcare decisions [34]. In agreement with the literature, our survey revealed that a website of this kind would be appreciated, in addition to paper-based materials, because it could be continually updated with information on a wide range of topics [21]. For this reason, we recently decided to go further with the improvement of the website, “www.dietachetogenica.unimore.it” (accessed on 31 March 2021), to offer free selective informative material about the ketogenic diet.

Overall, our survey demonstrated a high degree of satisfaction among the survey participants with the management of the ketogenic diet by using web-based materials among the survey participants. Nevertheless, the study has some obvious limitations, mainly related to the time at which the study was conducted and then the current conditions that could have affected the results. Another limitation, not least in importance, is that 82% of survey participants were treated in one centre. In future, it will be necessary to increase the number of Italian centres included in the survey to make the results much more representative of Italy. On the other hand, the study could represent a cue for dietary management during the ongoing crisis, as well as after the coronavirus outbreak resolves.

## 5. Conclusions

Our survey showed that caregivers with free access to web-based materials were markedly more satisfied than those with access only to paper-based materials. Their management of the ketogenic diet appeared to be more successful when they had different options. Thus, we confirm that different kinds of e-health applications should be used simultaneously, as complementary resources, in the management of the ketogenic diet. Finally, we suggest that our study could serve as a starting point for the management of the ketogenic diet in Italy, during the ongoing COVID-19 pandemic and after it resolves.

## Data Availability

Data available on request because of the agreement signed with granting agency.

## Supplementary Materials

The following are available online at https://www.mdpi.com/article/10.3390/nu13041240/s1, Information satisfaction questionnaire (ISQ) on the caregiver informative materials about the ketogenic diet.

## References

[1] Blaya J.A., Fraser H.S.F., Holt B. (2010). E-Health Technologies Show Promise in Developing Countries. Health Aff..

[2] Ricci G., Baldanzi S., Seidita F., Proietti C., Carlini F., Peviani S., Antonini G., Vianello A., Siciliano G., Musumeci O. (2018). A Mobile App for Patients with Pompe Disease and Its Possible Clinical Applications. Neuromuscul. Disord..

[3] Semprino M., Fasulo L., Fortini S., Martorell Molina C.I., González L., Ramos P.A., Martínez C., Caraballo R. (2020). Telemedicine, Drug-Resistant Epilepsy, and Ketogenic Dietary Therapies: A Patient Survey of a Pediatric Remote-Care Program during the COVID-19 Pandemic. Epilepsy Behav..

[4] Lo M.D., Gospe S.M. (2019). Telemedicine and Child Neurology. J. Child. Neurol..

[5] Liu X., Wang R., Zhou D., Hong Z. (2016). Feasibility and Acceptability of Smartphone Applications for Seizure Self-Management in China: Questionnaire Study among People with Epilepsy. Epilepsy Behav..

[6] Dorsey E.R., Glidden A.M., Holloway M.R., Birbeck G.L., Schwamm L.H. (2018). Teleneurology and Mobile Technologies: The Future of Neurological Care. Nat. Rev. Neurol..

[7] Kossoff E.H., Turner Z., Adams J., Bessone S.K., Avallone J., McDonald T.J.W., Diaz-Arias L., Barron B.J., Vizthum D., Cervenka M.C. (2020). Ketogenic Diet Therapy Provision in the COVID-19 Pandemic: Dual-Center Experience and Recommendations. Epilepsy Behav..

[8] Kondylakis H., Katehakis D.G., Kouroubali A., Logothetidis F., Triantafyllidis A., Kalamaras I., Votis K., Tzovaras D. (2020). COVID-19 Mobile Apps: A Systematic Review of the Literature. J. Med. Internet Res..

[9] Ross J., Stevenson F., Lau R., Murray E. (2016). Factors That Influence the Implementation of E-Health: A Systematic Review of Systematic Reviews (an Update). Implement. Sci..

[10] Pandher P.S., Bhullar K.K. (2016). Smartphone Applications for Seizure Management. Health Inform. J..

[11] Le S., Shafer P.O., Bartfeld E., Fisher R.S. (2011). An Online Diary for Tracking Epilepsy. Epilepsy Behav..

[12] Zarnowska I.M. (2020). Therapeutic Use of the Ketogenic Diet in Refractory Epilepsy: What We Know and What Still Needs to Be Learned. Nutrients.

[13] Wells J., Swaminathan A., Paseka J., Hanson C. (2020). Efficacy and Safety of a Ketogenic Diet in Children and Adolescents with Refractory Epilepsy—A Review. Nutrients.

[14] Martin-McGill K.J., Bresnahan R., Levy R.G., Cooper P.N. (2020). Ketogenic Diets for Drug-Resistant Epilepsy. Cochrane Database Syst. Rev..

[15] Pasca L., Varesio C., Ferraris C., Guglielmetti M., Trentani C., Tagliabue A., Veggiotti P., De Giorgis V. (2020). Families’ Perception of Classic Ketogenic Diet Management in Acute Medical Conditions: A Web-Based Survey. Nutrients.

[16] Zini E.M., Tagliabue A., Trentani C., Ferraris C., Boninsegna R., Quaglini S., Lanzola G. (2018). An MHealth Application for Educating and Monitoring Patients Treated with a Ketogenic Diet Regimen. Stud. Health Technol. Inf..

[17] Marchiò M., Roli L., Lucchi C., Costa A.M., Borghi M., Iughetti L., Trenti T., Guerra A., Biagini G. (2019). Ghrelin Plasma Levels After 1 Year of Ketogenic Diet in Children with Refractory Epilepsy. Front. Nutr..

[18] Marchiò M., Roli L., Giordano C., Trenti T., Guerra A., Biagini G. (2018). Decreased Ghrelin and Des-Acyl Ghrelin Plasma Levels in Patients Affected by Pharmacoresistant Epilepsy and Maintained on the Ketogenic Diet. Clin. Nutr..

[19] Leone A., De Amicis R., Lessa C., Tagliabue A., Trentani C., Ferraris C., Battezzati A., Veggiotti P., Foppiani A., Ravella S. (2019). Food and Food Products on the Italian Market for Ketogenic Dietary Treatment of Neurological Diseases. Nutrients.

[20] Ferraris C., Guglielmetti M., Tamagni E., Trentani C., De Giorgis V., Pasca L., Varesio C., Ferraro O.E., Tagliabue A. (2020). Use of Remote Monitoring by E-Mail for Long-Term Management of the Classic Ketogenic Diet. Nutrients.

[21] Cavalieri S., Marchiò M., Bondi M., Biagini G. (2019). Assessing Caregiver Informative Materials on the Ketogenic Diet in Italy: A Textual Ethnographic Approach. Token.

[22] Karakis I., Cole A.J., Montouris G.D., San Luciano M., Meador K.J., Piperidou C. (2014). Caregiver Burden in Epilepsy: Determinants and Impact. Epilepsy Res. Treat..

[23] Loblaw D.A., Bezjak A., Bunston T. (1999). Development and Testing of a Visit-Specific Patient Satisfaction Questionnaire: The Princess Margaret Hospital Satisfaction with Doctor Questionnaire. J. Clin. Oncol..

[24] Weissenstein A., Straeter A., Villalon G., Luchter E., Bittmann S. (2011). Parent Satisfaction with a Pediatric Practice in Germany: A Questionnaire-Based Study. Ital. J. Pediatrics.

[25] Lin K.-L., Lin J.-J., Wang H.-S. (2020). Application of Ketogenic Diets for Pediatric Neurocritical Care. Biomed. J..

[26] Kossoff E.H., Zupec-Kania B.A., Auvin S., Ballaban-Gil K.R., Christina Bergqvist A.G., Blackford R., Buchhalter J.R., Caraballo R.H., Cross J.H., Dahlin M.G. (2018). Optimal Clinical Management of Children Receiving Dietary Therapies for Epilepsy: Updated Recommendations of the International Ketogenic Diet Study Group. Epilepsia Open.

[27] Testa F., Marchiò M., Belli M., Giovanella S., Ligabue G., Cappelli G., Biagini G., Magistroni R. (2019). A Pilot Study to Evaluate Tolerability and Safety of a Modified Atkins Diet in ADPKD Patients. PharmaNutrition.

[28] Rezaei S., Abdurahman A.A., Saghazadeh A., Badv R.S., Mahmoudi M. (2019). Short-Term and Long-Term Efficacy of Classical Ketogenic Diet and Modified Atkins Diet in Children and Adolescents with Epilepsy: A Systematic Review and Meta-Analysis. Nutr. Neurosci..

[29] Kossoff E., Cervenka M. (2020). Ketogenic Dietary Therapy Controversies for Its Second Century. Epilepsy Curr..

[30] Costa A.-M., Lucchi C., Malkoç A., Rustichelli C., Biagini G. (2021). Relationship between Delta Rhythm, Seizure Occurrence and Allopregnanolone Hippocampal Levels in Epileptic Rats Exposed to the Rebound Effect. Pharmaceuticals.

[31] Lucchi C., Marchiò M., Caramaschi E., Giordano C., Giordano R., Guerra A., Biagini G. (2017). Electrographic Changes Accompanying Recurrent Seizures under Ketogenic Diet Treatment. Pharmaceuticals.

[32] Goswami J.N., Sharma S. (2019). Current Perspectives on The Role of The Ketogenic Diet in Epilepsy Management. Neuropsychiatr. Dis. Treat..

[33] Li H., Jauregui J.L., Fenton C., Chee C.M., Bergqvist A.G.C. (2014). Epilepsy Treatment Simplified through Mobile Ketogenic Diet Planning. J. Mob. Technol. Med..

[34] Tao D., LeRouge C., Smith K.J., De Leo G. (2017). Defining Information Quality into Health Websites: A Conceptual Framework of Health Website Information Quality for Educated Young Adults. JMIR Hum. Factors.

