# Supplementary material for: Evaluation of E-Health Applications for Paediatric Patients with Refractory Epilepsy and Maintained on Ketogenic Diet
Source: Nutrients. 2021 Apr 9;13(4):1240. doi: 10.3390/nu13041240 (PMC8069190; doi:10.3390/nu13041240)
Supplement: Supplementary file 1 [file nutrients-13-01240-s001.pdf]

Information satisfaction questionnaire (ISQ) on the caregiver informative materials about the ketogenic diet

FIRST SECTION – General information

1. **Gender and age of the survey participant:**
- Female
- Male
- Both parents are participating in the survey

Age range:

- 30-40
- 40-50
- 50-60
- >60

2. **Number of children in the family:**
- 1
- 2
- 3
- >3

3. **Age of the child on the ketogenic diet:**
- 0-3 years old
- 4-6 years old
- 7-9 years old
- 10-13 years old
- 14-17 years old

4. **Gender of the child on the ketogenic diet:**
   - Male
   - Female
5. **Name of the hospital tracking the child:**
   - Pavia
   - Roma
   - Bologna
   - Firenze
   - Modena
6. **Is your child still treated with the ketogenic diet?**
   - Yes → duration of the treatment: ______________
   - No, my child was treated with the ketogenic diet in the past → duration of the treatment: ____
7. **Who is/was the person in charge of implementing the diet at home?**
   - Mother
   - Father
   - Both
   - ______________

8. Do you have access to the internet?

- Yes, always
- Yes, at least once a week
- Yes, sometimes
- No

SECOND SECTION – Where did you find preliminary information about the ketogenic diet?

1. **Where did you find preliminary information about this diet? (choose one or more options):**
   - Websites
   - Social Media
   - Hospital/health professionals
   - Word of mouth
   - Booklets/leaflets
   - Other (please specify)

2. **Where did you find preliminary information about the hospitals offering the ketogenic diet protocol, before contacting a specialized centre? (choose one or more options):**
   - Websites
   - Social Media
   - Hospital/health professionals
   - Word of mouth
   - Booklets/leaflets
   - Other (please specify)

3. **Which kind of informative materials were provided by the hospital where your children were/are under treatment? (choose one or more options):**
   - Paper-based materials produced by the hospital
   - Paper-based materials produced by pharmaceutical companies
   - Website
   - App
   - Videos
   - Other (please specify)

THIRD SECTION – Can you evaluate the informative materials on the ketogenic diet given by the centre that follows your child?

How much are you IN AGREEMENT or DISAGREEMENT with the following sentences? Circle a number from 1 (strongly disagree) to 5 (strongly agree).

	**INFORMATIVE MATERIAL**	Strongly disagree	Disagree	I do not Know	Agree	Strongly Agree
**Booklets/leaflets**	The booklets/leaflets are readable	1	2	3	4	5
	The booklets/leaflets include comprehensive information	1	2	3	4	5
	The booklets/leaflets include all the preliminary data to help choose the treatment	1	2	3	4	5
	The booklets/leaflets include all the information for the management of the diet (recipes, etc.)	1	2	3	4	5
	The booklets/leaflets include all the information about the benefits of the diet (in the short and long term)	1	2	3	4	5
	The booklets/leaflets include all the information about the side effects of the diet (in the short and long term)	1	2	3	4	5
	The booklets/leaflets include all the information about when and how to call specialists in case of need	1	2	3	4	5
	The booklets/leaflets are useful tools in motivating families to choose the ketogenic diet	1	2	3	4	5
	My confidence in the dietary treatment has increased after having access to this informative material	1	2	3	4	5

**Website**	The website includes comprehensive information	1	2	3	4	5
	The graphic image on the website facilitates access to the information	1	2	3	4	5
	The website is easy to navigate	1	2	3	4	5
	The website contains adequate language to be understood by people of different educational levels	1	2	3	4	5
	The website includes all the preliminary data to help choose the treatment	1	2	3	4	5
	The website includes all the information for the management of the diet (recipes, etc.)	1	2	3	4	5
	The website includes all the information about the benefits of the diet (in the short and long term)	1	2	3	4	5
	The website includes all the information about the side effects of the diet (in the short and long term)	1	2	3	4	5
	The website includes all the information about when and how to call specialists in case of need	1	2	3	4	5
	The website is a useful tool in motivating families to choose the ketogenic diet	1	2	3	4	5
	My confidence in the dietary treatment has increased after having access to this informative material	1	2	3	4	5

**App**	The app includes readable information	1	2	3	4	5
	The app includes comprehensive information	1	2	3	4	5
	The graphic image on the app facilitates access to the information	1	2	3	4	5
	The app is easy to navigate	1	2	3	4	5
	The app is easy to use with different devices (smartphone, tablet, and computer)	1	2	3	4	5
	The app contains adequate language to be understood by people of different educational levels	1	2	3	4	5
	The app facilitates dietary management in everyday life	1	2	3	4	5
	The app facilitates dietary management in public places	1	2	3	4	5
	The app includes all the information about when and how to call specialists in case of need	1	2	3	4	5
	The app is a useful tool in motivating families to choose the ketogenic diet	1	2	3	4	5
	My confidence in the dietary treatment has increased after having access to this informative material	1	2	3	4	5
**Overall material**	I am overall satisfied with the informative materials provided by the hospital	1	2	3	4	5
	The informative materials have changed our attitude on the treatment in a positive way	1	2	3	4	5
	The informative material has made us become more aware of the management of the diet	1	2	3	4	5
	The informative materials has stimulated us to continue with the diet	1	2	3	4	5
	The information received has helped us explain the ketogenic diet to our children	1	2	3	4	5
	The information received has helped us to explain the ketogenic diet to other people involved in the management of the diet	1	2	3	4	5
	I would use the informative materials to help another family choose the ketogenic diet.	1	2	3	4	5

FOURTH SECTION – Suggestions to improve the information for families

1. **What would you suggest improving in the materials you had received?**

2. **What extra information would you have liked to receive?**

3. **What was the most and the least useful thing in the informative texts you were given?**

4. **What medium of communication did you consider the most valuable among those cited in the questionnaire?** (website, app, videos, paper-based materials produced by the hospital, and paper-based materials produced by pharmaceutical companies)

*This is the translated version of the Italian questionnaire published by Cavalieri et al. (2019).
